# Two Quantum Protocols for Oblivious Set-member Decision Problem

**DOI:** 10.1038/srep15914

**Published:** 2015-10-30

**Authors:** Run-hua Shi, Yi Mu, Hong Zhong, Jie Cui, Shun Zhang

**Affiliations:** 1 School of Computer Science and Technology, Anhui University, Hefei City, 230601, China; 2 Centre for Computer and Information Security Research, School of Computing and Information Technology, University of Wollongong, Wollongong NSW 2522, Australia

## Abstract

In this paper, we defined a new secure multi-party computation problem, called Oblivious Set-member Decision problem, which allows one party to decide whether a secret of another party belongs to his private set in an oblivious manner. There are lots of important applications of Oblivious Set-member Decision problem in fields of the multi-party collaborative computation of protecting the privacy of the users, such as private set intersection and union, anonymous authentication, electronic voting and electronic auction. Furthermore, we presented two quantum protocols to solve the Oblivious Set-member Decision problem. Protocol I takes advantage of powerful quantum oracle operations so that it needs lower costs in both communication and computation complexity; while Protocol II takes photons as quantum resources and only performs simple single-particle projective measurements, thus it is more feasible with the present technology.

Cryptography is an important tool that enables the secure transmission of a secret message between a sender and a recipient from any potential eavesdropper. On the one hand, however, the security of most classical cryptosystems is based on the assumption of computational complexity, which is strongly challenged by the increasing capability of computations or algorithms^1^,^2^. Especially, it is believed that some mathematical difficulties, e.g. the integer factorization or the discrete logarithm problems, may be fragile in the future with the presence of quantum computers. On the other hand, fortunately, this difficulty can be overcome by quantum cryptography^3^,^4^, where the security is guaranteed by physical principles. Since Bennett and Brassard presented the first quantum key distribution protocol^5^, quantum cryptography has been widely studied and rapidly developed. Accordingly, a lot of results have been gained, such as quantum key distribution^6^, quantum teleportation^7^, quantum signature^8^, and other novel quantum computations^9^.

Furthermore, in many cryptographic tasks, it requires to protect not only the data privacy, but also the user privacy. Private query is an important problem of this type. Suppose that a user, Alice, wants to know an item of a database held by a database provider, Bob, but does not want him to know which item she is interested in. Bob in turn wants to limit the amount of item that she can get from the database.

In 2008, Giovannetti, *et al*.^10^,^11^ for the first time presented a cheat sensitive quantum private query (QPQ) protocol. In their protocol, Alice and Bob only exchange two quantum messages. For example, Alice wants to find out the *j*th record of Bob’s database. She first prepares two *n*-qubit query states 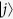 and 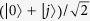. She then sends, in random order, these two query states to Bob, waiting for his first reply before sending the second. As a response to query, Bob performs two oracle operations on the two query states and then sends them back to Alice, respectively. Finally, Alice processes the two returned states 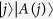 and 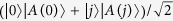, where the 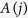 is the content of the *j*th record in the database. By measuring the first state she obtains the value of 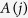, and further she checks Bob’s potential attack with 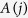, that is, she checks whether the superposition in the second state is preserved. Compared to known private information retrieval protocols, this QPQ protocol achieves an exponential reduction in both communication and computation complexity. Later, Olejnik^12^ presented an improved protocol for QPQ using phase-encoded queries, in which the oracle operation and the encoding method are subtly selected so that one query state 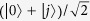 can achieve two aims simultaneously, i.e., obtaining the expected item and checking Bob’s potential attack. So the communication complexity and the computation complexity in Olejnik’s protocol are further reduced.

In addition, Jakobi *et al.*^13^ proposed a novel and practical quantum private query protocol based on SARG04 quantum key distribution (QKD) protocol^14^. By using SARG04 QKD, an asymmetric key can be distributed between Alice and Bob, where Alice only knows one bit of the key, while Bob knows the whole key. For instance, Bob prepares a long sequence of photons which are randomly in one of four states 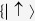, 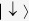, 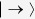, 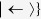 and sends them to Alice. Then Alice measures each received photon in 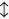 or 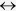 basis at random. Obviously, Alice will measure half of the qubits she receives in the correct basis. When Bob subsequently announces the bases, we can easily see that (I) Bob knows the entire “raw key”, (II) Alice knows half of the bits and (III) Bob cannot know which ones Alice has measured correctly. In order to reduce Alice’s information on the key, Alice and Bob cut the raw key into multiple substrings of length *N*, and add these strings bitwise to obtain the final key with length *N*. Later, Gao *et al.* generalized Jakobi’s protocol and proposed a similar 4-state QPQ protocol^15^, which uses four generalized states 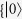, 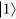, 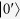, 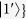, where 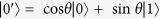 and 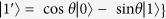. Gao’s protocol exhibits better database security than Jakobi’s protocol, but has a higher probability with which Bob can correctly guess the address of Alice’s query. Subsequently, to improve the security, yang *et al.* proposed a flexible B92-based QPQ protocol^16^.

In this paper, we define a new but interesting problem, Oblivious Set-member Decision problem, which allows a server, Bob, to decide whether a secret of a user, Alice, belongs to his private set in an oblivious manner. That is, Bob wants to know whether Alice’s secret is a member of his private set. But Alice does not want him to know which member it is. Oblivious Set-member Decision can be used to privately compute multi-party set intersection and union which are widely applied in some privacy-preserving and information-sharing settings^17^. In addition, there are also lots of practical applications of Oblivious Set-member Decision in fields of the identifiable or verifiable circumstances, such as anonymous authentication, electronic voting and electronic auction. Thus Oblivious Set-member Decision problem is one of the most fundamental and key problems within the multi-party collaborative computation of protecting the privacy of the users.

In next section, inspired by the QPQ protocols mentioned above, we proposed two quantum protocols for Oblivious Set-member Decision problem, which one subtly applies the powerful quantum oracle operations, while the other utilizes the asymmetric key between Alice and Bob based on the technologies of Quantum Key Distribution.

## Results

Here, we first give a definition of Oblivious Set-member Decision protocol.

**Definition 1** (Oblivious Set-member Decision Protocol). A user, Alice, inputs a secret *k*, and a server, Bob, inputs a private set 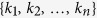. Finally, Alice gets nothing but Bob outputs one bit 0 or 1. This protocol should meet the following requirements:

### Correctness

Bob gets 1 if *k* ∈{*k*_1,_ *k*_2, …_ *k*_n_}, and 0 otherwise.

### Alice’s Privacy

Except knowing whether Alice’s secret belongs to his private set, Bob cannot obtain any other secret information about Alice’s secret *k*.

### Alice’s anonymity

Bob cannot know which member it is, if Alice’s secret is a member of his private set.

### Bob’s Privacy

Alice cannot know any secret information about Bob’s private set.

### Protocol I

Protocol I follows some ideas from QPQ in refs 10,12, and refers to the oracle of Grover search algorithm^2^. Suppose Alice’s secret *k* and Bob’s all private member *k*_*i*_s are the elements of 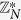, where 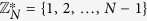. Protocol I consists of 5 steps, which are described in detail as follows:

**Step 1**. Alice prepares a query state 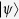, where 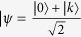 and *k* is her secret. Then Alice sends the query state 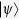 to Bob by an authenticated quantum channel.

**Step 2**. After receiving the query state 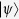 from Alice, Bob applies an oracle *O*_1_ on it, where the oracle *O*_1_ is a unitary operator, defined as follows:


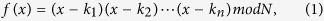



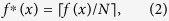



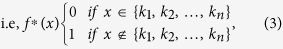



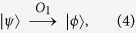



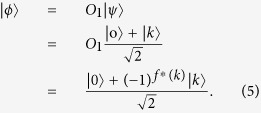


Furthermore, Bob tosses a coin to decide whether applies another oracle 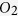 on the state 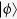. That is, if the outcome is the head, he performs the oracle 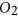 on the state 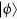. Otherwise, he does nothing. Obviously, he performs the oracle 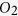 on the state 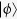 only with the probability of 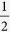, where the oracle 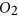 is defined by


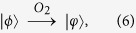



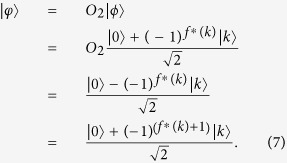


Then Bob sends the state 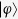 back to Alice by the authenticated quantum channel.

**Step 3**. After receiving the state 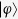 from Bob, Alice performs an honest test. That is, Alice checks whether the superposition in the returned state is preserved as follows: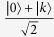 or 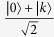. Since the two possible states are obviously orthogonal and further Alice knows the value of *k*, she is able to completely distinguish them by a von Neumann measurement. If Alice finds a cheat of Bob, she will terminate this protocol; otherwise continue to the next step.

**Step 4**. Alice extracts out the phase information 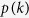 of the returned state 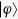 by distinguishing it between 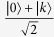 and 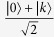, i.e., 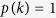 if it is in the state 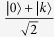, and 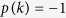 otherwise. Then she sends the classical information 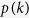 to Bob by the authenticated classical channel.

**Step 5**. After receiving the classical information 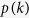 from Alice, Bob decrypts it to further obtain 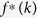, where 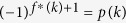 if he has applied the oracle 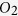, and 
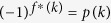
 otherwise. If 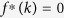, Bob can decide that Alice’s secret belongs to his private set. Otherwise, it doesn’t.

**Protocol II.**

Protocol II is inspired by the ideas from refs 13,15,16 in which an asymmetric key is distributed between Alice and Bob based on Quantum Key Distribution, where Alice only knows a few bits of the key, while Bob knows the whole key. Protocol II includes 6 steps, which is described in detail as follows:

**Step 1**. Bob creates an *N*-element database by his private set 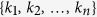, where the *j*th element 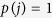 if 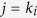 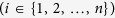, and 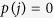 otherwise. Furthermore, Bob generates a random integer 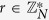 and computes 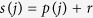 for 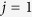 to *N* (encryption). Here + denotes the binary XOR operation.

**Step 2**. By calling Gao *et al.*’s protocol^15^, Alice and Bob share an *N*-bit key 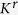, where Bob knows the whole key 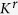 and Alice knows only *q* bits of 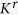, where *q* is a security parameter. Furthermore, among these *q* bits, Alice randomly chooses 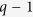 bits to check Bob’s honesty. That is, she requests Bob to announce the values of these bits. If these bits announced by Bob aren’t completely same as those Alice has recorded, it will show that Bob is dishonest. If Alice finds a cheat of Bob, she will terminate this protocol; otherwise continue to the next step.

**Step 3**. Suppose the remaining one bit known by Alice is the *j*th bit 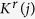 of the raw key 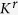. However, she expects to get the *k*th bit of the shared key, where *k* is Alice’s secret. So she declares the number 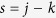.

**Step 4**. Bob replaces the announced 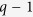 bits in the key 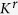 by random 0 or 1 integer. Then Bob shifts 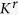 by *s* and finally gets an asymmetric key *K* shared between Alice and Bob, where Bob knows the whole shared key, while Alice only knows the *k*th bit of the shared key. Furthermore, Bob encrypts all 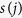
s by using the key *K* in one-time pad method, that is, he computes 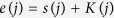 for 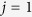 to *N*, where 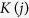 is the *j*th bit of the shared key *K*. Then Bob publishes the whole encrypted database (i.e., all 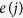
s for 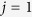 to *N*) at a public server.

**Step 5**. Alice gets 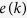 from the public encrypted database of Bob, and further decrypts it to obtain 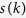, since 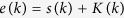 and she (only) knows 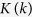. Then Alice sends the classical information 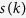 to Bob by the authenticated classical channel.

**Step 6**. After receiving the classical information 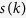 from Alice, Bob computes 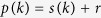 (decryption). If 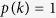, then he can deduce that Alice’s secret *k* belongs to his private set 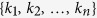, i.e., 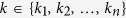. Otherwise, 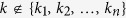.

**Figure 1:**
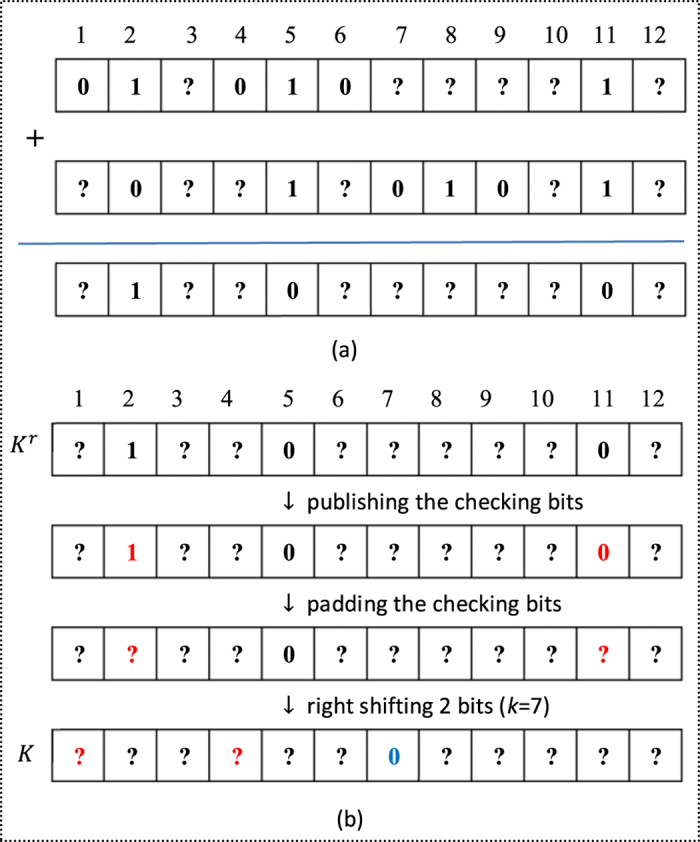
Illustration of creating the asymmetric key. (**a**) How to reduce Alice’s information on the key. (**b**) How to process the raw key 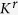 to get the final key *K*.

**Figure 2:**
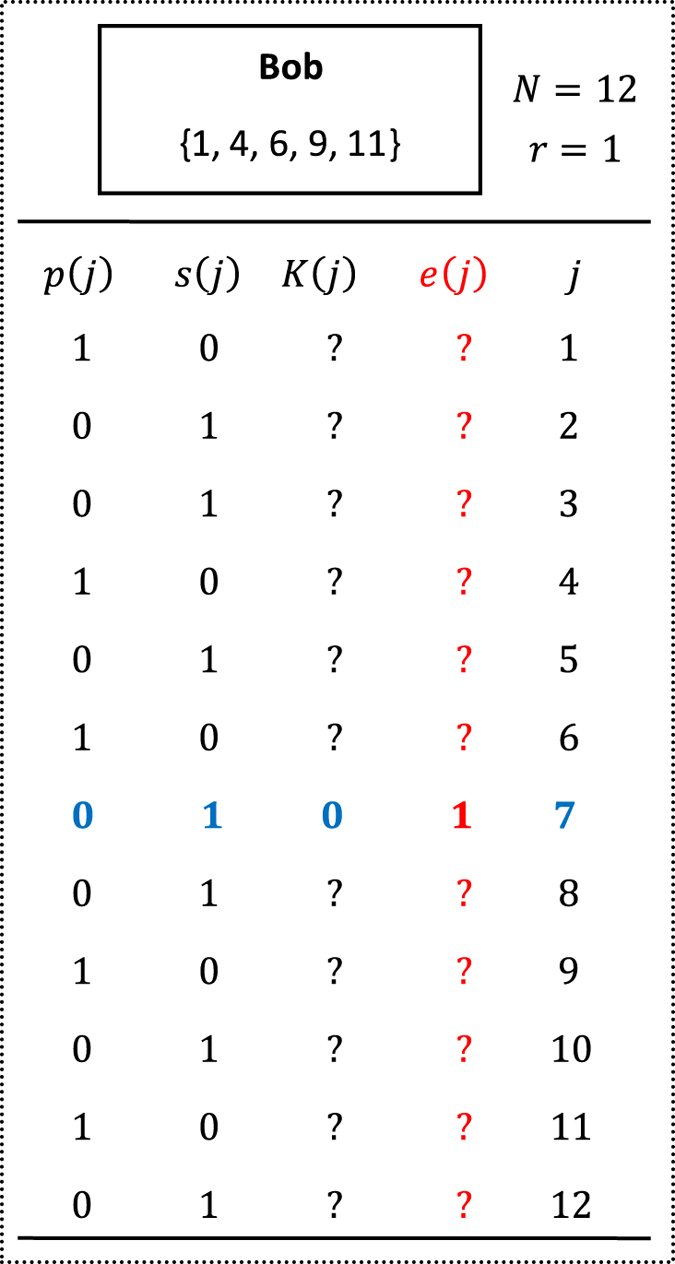
Bob’s encoding and encrypting methods. All 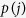
s, 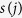
s and 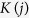
s are private, while all 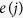
s are public. Alice can only decrypt 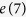 to further get 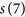 with the key 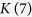. Finally, Bob can rightly get 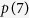 by computing 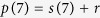.

Here we give a simple example to better illustrate Protocol II, as shown in Figs 1 and 2. In our example, Alice has a secret 7 (i.e., *k* = 7), and Bob has a private set 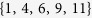 in 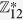. On the one hand, Alice and Bob share an asymmetric key *K* (see Fig. 1), where Alice only knows the seventh bit of *K* (i.e., *K*(7)), while Bob knows all bits of *K*. On the other hand, Bob creates a private database 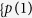, 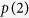,…, 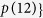 by his private set, where 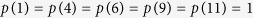 and other 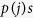 are equal to 0 (see Fig. 2), further encrypts each item 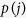 twice by using two different keys, *r* and 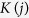, and finally publishes all 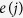
s. Obviously, Alice can rightly get 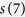 by computing 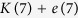, but not 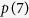 without knowing *r*. However, Bob can rightly get 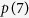 by computing 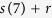, but he does not know which item of his private database it is equivalent to, except knowing Alice’s secret does not belong to his private set since 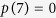.

## Security of the protocols

**Protocol I.** The oracles 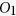 and 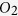 are all phase transformation operations, where the former is utilized to encode 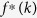, while the later to further encrypt it in the one-time pad method. On the one hand, obviously Alice doesn’t know 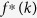 with 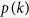 due to the oracle 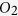. However, Bob can get it rightly by whether or not the oracle 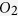 has been performed and then he can easily decide whether Alice’s secret lies in his private set by the value of 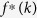. That is, it guarantees the **correctness** of Protocol I. On the other hand, we can easily see that 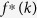 doesn’t leak Alice’s secret *k*. Even if 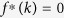, it doesn’t yet leak which member it is equal to. That is, it guarantees **Alice’s anonymity**.

Furthermore, **Alice’s privacy** depends on Bob’s impossibility of discriminating the query state sent from Alice. Two basic laws of quantum theory enforce this: No-cloning Theorem which forbids the creation of identical copies of an arbitrary unknown quantum state, and Heisenberg Uncertainty Principle which implies that it is impossible to measure the state of any system without disturbing that system. In order to extract the secret information about *k* from the query state 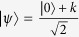, obviously Bob must measure the state 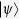, but he will certainly disturb it. We will analyze two measure-based attacks by a dishonest Bob in detail.

First, if Bob directly measures the query state 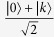 by a simple projective measurement (intercept), the measured result can be either 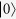 or 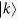 with the probabilities 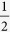 and 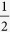, respectively. If he gets 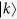, he can successfully pass the honest test by re-preparing a new quantum system in the state 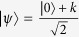 and returning it to Alice (resend). However, if he gets 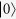, he cannot pass the honest test. In short, this intercept-resend attack will be discovered in the honest test with the probability of 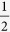. That is, Protocol I is cheat sensitive^10^,^12^.

Furthermore, we discuss a more complicated entangle-measure attack by a dishonest Bob that he is able to prepare an ancillary system and entangle the ancillary system with the query state from Alice by his local unitary operations, and afterwards he can measure the ancillary system to get the partial information about Alice’s secret. Suppose that the initial state of the ancillary system is 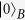 and Bob’s dishonest action when he receives Alice’s query state can be described by a unitary operator 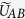 as follows:


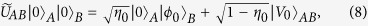



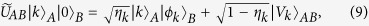



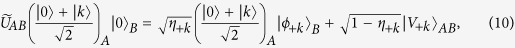


where 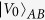, 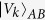 and 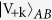 are the vector orthogonal to 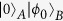, 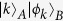 and 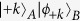 
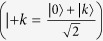
, respectively, i.e.,


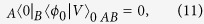



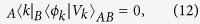



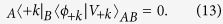


In order to completely pass the honest test, we can easily deduce that the following condition holds in Eq. (10):


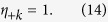


That is,


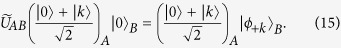


In addition, after applying the unitary operator 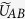, in order to fully pass the honest test, the returned states cannot contain other vectors except the vectors of 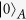 and 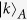. So Eqs. (8) and (9) should be changed into the following equations, accordingly:


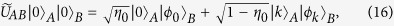



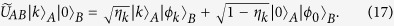


By Eq. (15), when 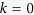, we further get


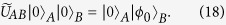


That is,


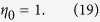


In addition, we can get


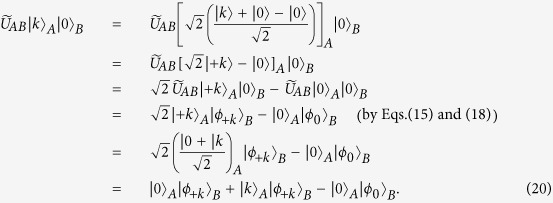


If we compute the scalar product between Eqs. (17) and (20), then we will obtain the identity


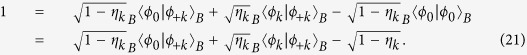


Since 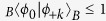 and 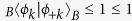, so we get


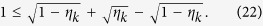


That is,


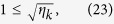


which implies


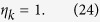


Thus, we can obtain the following expanded expression


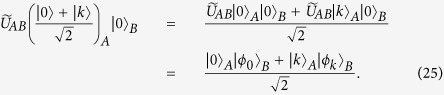


Similarly, if we compute the scalar product between Eqs. (15) and (25), then we will obtain


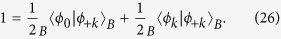


By Eq. (26), it gives


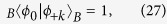



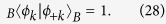


From Eqs. (27) and (28), it shows that if Bob wants to be sure that he passes the honest test, then the final states of the ancillary system *B* for any choice of *k* will coincide with 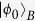, that is, the states of the ancillary system *B* are independent from the secret *k*. Therefore, even though Bob performs an entangle-measure attack, he will not obtain any secret information about the secret *k*.

In addition, **Bob’s privacy** is guaranteed by the encoding and encrypting methods discussed above. If Alice honestly executes this protocol, she cannot obtain any secret information about Bob’s private set. If Alice is dishonest, the simplest attack for her is to send a false query state 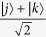 to Bob, instead of the true query state 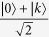. Then the corresponding state returned from Bob will be in 
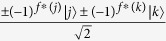
 (i.e., 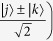. From the returned state, Alice can only infer that 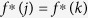 or 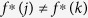, but she cannot further deduce whether *j* or *k* belongs to Bob’s private set because she does not know the values of 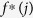 and 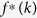. Furthermore, for more general case, Alice sends a more general sate 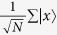 to Bob, instead of the true query state 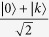. Accordingly, the returned state will be in 
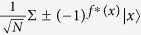
, where 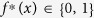. Obviously, Alice cannot extract out the phase information 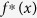 of single basis state 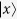 from the returned state, though she can approximatively count the number of the members in Bob’s private set. However, if Alice sends a false query state, she will run a risk with the probability of 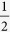 that she cannot gain 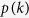 rightly, which further affects Bob’s right output. That is, Bob cannot rightly make a decision of the set-member relation without the right phase information 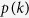. For example, in anonymous authentication applications, If Alice can prove that her secret is a member of Bob’s private set (but which member is unknown) by Protocol I, then Bob will believe that Alice is an authorized user and further open the corresponding resources or provide services to Alice. But, if Alice sends a false query state, the verification will fail with the probability of 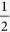.

### Protocol II.

When Alice and Bob honestly execute this protocol, the **correctness** is guaranteed by the asymmetric key shared between Alice and Bob, whose security is based on the security of Quantum key Distribution^18^,^19^,^20^.

In Protocol II, Alice only sends the classical messages *s* and 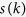 to Bob except checking information. Clearly, Bob cannot get any secret information about Alice’s secret only from these messages except knowing whether it is a member of his private set. That is, it guarantees **Alice’s privacy**. Furthermore, **Alice’s anonymity** depends on the security of the asymmetric key^13^,^15^. When creating the asymmetric key, if Bob is dishonest, he can perform the following two attacks: one is to send other states (e.g., 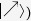 than he announces (e.g., 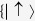, 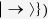, and the other is to perform an entangle-measure attack, that is, he prepares a state of two qubits 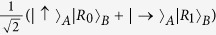, where the first qubit is sent to Alice and the second is kept in Bob’s register, and afterwards he will measure the state in his register to gain some information on the conclusiveness of Alice’s measurement. However, as analyzed in refs 13,15 these attacks will introduce bit errors. That is, if Bob gains information on the conclusiveness of Alice’s bits, he will lose information on the bit values she has recorded. In fact, it is impossible for Bob to have both the correct bit value and conclusiveness information of Alice’s measurement (i.e., the address of the correct basis). Therefore, Bob cannot simultaneously obtain the bit 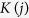, which is the correctly measured result of Alice, and the corresponding address *j*. In our proposed Protocol II, in order to check Bob’s honesty, Alice will compare 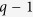 measurement results with these corresponding bits that Bob’s announces. Thus, for a dishonest Bob, the success probability to completely pass the honest test in Step 2 of Protocol II is not more than 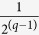.

We further analyze **Bob’s privacy**. On the one hand, if Alice is dishonest and she wants to obtain more items (i.e., 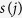
s) in Bob’s private database, she has to try to obtain more bits of the shared key. As analyzed in refs 13,15 it is possible for a dishonest Alice to store the qubits received from Bob and then take more effective measurements (e.g., the optimal unambiguous state discrimination (USD) measurement) on them after getting Bob’s public information. Even so, the advantage Alice obtains by USD measurement is negligible compared with the honest measurement^13^,^15^. On the other hand, though a dishonest Alice can theoretically get more than one 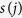, she doesn’t yet know any 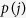 rightly since 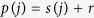 and *r* is unknown. By these 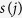
s, she can only decide that these indexes can be roughly classified into at most two categories: one belongs to Bob’ private set and the other doesn’t belong to it. But she cannot decide which category belongs to Bob’s private set.

We have analyzed the security of proposed protocols. However, please note that we mainly consider the honest-but-curious parties^21^ in our protocols, which is similar to the semi-honesty model in the classical settings. In classical settings, any secure protocol in semi-honesty model can be correspondingly translated into a secure protocol in malicious model. However, it still needs to further study how to translate a protocol from semi-honesty model to malicious model in quantum settings. It is also our future work (especially, the definition of malicious model in quantum settings).

### Performance Comparisons

Here, we give a simple comparison of our proposed protocols with the related QPQ protocols. In Protocol I, we follow some ideas from QPQ in refs 10,12 to introduce two quantum oracles. However, compared to these related QPQ protocols, the oracles proposed in Protocol I are more specific and more elaborated, where one is for encoding, and the other is for encrypting. In Protocol II, we are inspired by the asymmetric key of QPQ in refs 13,15,16. However, compared to these related QPQ protocols, there are at least two good advantages of Protocol II: (1) When creating the asymmetric key, Alice knows some bits of the raw key, not just one. On the one hand, it is easier to control and create the raw key with the present technology. On the other hand, Alice can check the honesty of Bob by using the remaining bits among these known bits except one bit as the final key. (2) Bob cleverly creates a 0/1 database and further encrypts it twice by using different keys, thus it is more secure. Even if Alice knows more than one bit of the final asymmetric key, she also only knows the corresponding encrypted items.

Furthermore, we evaluate the performance of the proposed protocols, as listed in Table 1. In Protocol I, we introduce two powerful quantum oracle operations. In fact, the main operations of Protocol I are just the two oracle operations. In addition, Protocol I is a 3-round protocol obviously, which consumes 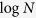 qubits quantum resource and 1 bit classical resource, and further performs a von Neumann measurement in *N*-dimensional Hilbert space. Thus, Protocol I needs only 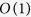 computation costs and 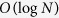 communication costs. For Protocol II, though its round is more than 3, it is also constant, irrespective of 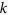, *n* and *N*. In Protocol 2, obviously it consumes 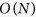 qubits to create the asymmetric key between Alice and Bob, and 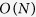 bits to store the classical database for Bob. In addition, Alice performs 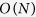 projective measurements in 2-dimensional Hilbert space and Bob computes 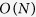 encryptions of one-time pad. So, Protocol II needs 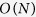 costs in both communication and computation complexity.

## Discussion

In this paper, we first defined Oblivious Set-member Decision problem and further proposed two constant round quantum protocols to solve the Oblivious Set-member Decision problem, where Protocol I has better advantages in term of communication and computation complexity due to powerful quantum oracle operations, while Protocol II takes photons as quantum resources and performs single-photon projective measurements, and thus it is more feasible with the present technology, that is, it is easier to implement it.

## Additional Information

**How to cite this article**: Shi, R.-h. *et al.* Two Quantum Protocols for Oblivious Set-member Decision Problem. *Sci. Rep.* **5**, 15914; doi: 10.1038/srep15914 (2015).

## Figures and Tables

**Table 1:** Comparison of two proposed protocols.

	Classical resources	Quantum resources	Oracle operation	Quantum measurement	Communication Complexity	Computation Complexity	Round
Protocol I	1 bit	*O*(log*N*)qubits	*Y*	*VNM_N*	*O*(log*N*)	*O*(1)	*O*(1)
Protocol II	*O*(*N*)bits	*O*(*N*)qubits	*N*	*SPM_*2	*O*(*N*)	*O*(*N*)	*O*(1)

Note: *Y*, *N*, *VNM_N* and *SPM_*2 denote Yes, No, von Neumann Measurement in *N*-dimensional Hilbert space and simple Projective Measurement in 2-dimensional Hilbert space, respectively.
